# A physics-informed neural network based on mixed data sampling for solving modified diffusion equations

**DOI:** 10.1038/s41598-023-29822-3

**Published:** 2023-02-13

**Authors:** Qian Fang, Xuankang Mou, Shiben Li

**Affiliations:** Department of Physics, Wenzhou University, Wenzhou, 325035 Zhejiang China

**Keywords:** Physics, Chemical physics, Condensed-matter physics

## Abstract

We developed a physics-informed neural network based on a mixture of Cartesian grid sampling and Latin hypercube sampling to solve forward and backward modified diffusion equations. We optimized the parameters in the neural networks and the mixed data sampling by considering the squeeze boundary condition and the mixture coefficient, respectively. Then, we used a given modified diffusion equation as an example to demonstrate the efficiency of the neural network solver for forward and backward problems. The neural network results were compared with the numerical solutions, and good agreement with high accuracy was observed. This neural network solver can be generalized to other partial differential equations.

## Introduction

Partial differential equations (PDEs), especially second-order PDEs, have been extensively used in physics, engineering, finance, and other fields. Some simple PDEs can be solved analytically, but most complex PDEs rely on numerical solutions, which are usually divided into forward and backward problems. Common numerical methods include finite difference^1–6^, finite element^7–13^, and Lagrange multiplier^14–18^. These conventional methods have been extensively applied to solve forward PDEs in various practical problems. However, the deep neural network (DNN) provides another solution for complex nonlinear PDEs without the domain discretization used in numerical methods and is thus suitable for forward and backward problems^19–23^.

DNN is exhibiting major advancement in solving PDEs and has attracted increasing attention in various research areas due to its universal approximations^24–27^. However, a large amount of labeled data is usually required for training DNN-based models to solve PDEs, and such data are often unavailable in many physical applications. To overcome this disadvantage, researchers have proposed a novel DNN-based neural network called the physics-informed neural network (PINN) help to reduce the needed training time in a physics-informed manner; in this network, physics-informed loss functions are constructed based on PDE residuals^20,28–31^. Generally, residual design plays an important role in PINN approximations, so residual DNN is a highly effective type of neural network^32–34^. PINN can encode any underlying physics law such that the differential operators in the governing PDEs can approximated by automatic differentiation^35,36^. With such advantages, PINNs have been applied extensively to solve complex PDEs in various application areas in recent years^37–47^. On the one hand, an increasing number of studies are being conducted to examine the approaches for building improved PINN models by incorporating such models into other methods. For example, Karniadakis et al. introduced a systematic PINN model for the first time and presented a series of PINN variants, including Bayesian PINN^48^, fractional PINN^49^, extended PINN^50^, parareal PINN^51^, non-Newtonian PINN^52^, hp-variational PINN^53^, and nonlocal PINN^54^. These extended PINN models were constructed to approximate various forward PDEs, either linear or nonlinear, in application areas ranging from engineering to finance. On the other hand, PINN models have also been extended to backward problems, such as advection-dispersion equations^55^, stochastic problems^56^, flow problems^57^, and conservation laws^58^. In these backward problems, the training data are inputted into DNNs to screen the unknown parameters in the PDEs by constructing PINN loss functions.

In DNN, data sampling is another important factor for solving second-order or higher-order PDEs^21,59–61^. Latin hypercube sampling (LHS) filters the variance associated with the additive components of a transformation, and it is a powerful sampling method for data analysis in nearly every field of computational science, engineering, and mathematics^62–65^. In LHS, because the sample space is divided into a series of subspaces that are randomly paired, the LHS algorithm iterates to determine optimal pairings on the basis of some specified criteria. To solve PDEs using neural networks, LHS^28^ and simple random sampling (RS)^66,67^ can be used to sample data sets. LHS and RS are usually employed to solve PDEs in regular domains. However, other sampling methods based on domain decomposition or irregular domains have also been developed to solve PDEs^50,68,69^.

In this study, we developed an improved LHS method, namely, GLHS, where LHS and Cartesian grid sampling (GS) are merged and optimized to deal with a data set under the periodic boundary condition that commonly appears in the theory and simulation of polymer chains under bulk conditions. We are aware that in polymer physics, especially in self-consistent field theory (SCFT), the modified diffusion equation (MDE), which is a parabolic-type second-order PDE, is a key equation in SCFT for Gaussian and wormlike chains^70–72^. Several classical numerical methods have been developed to solve MDEs in SCFT and have achieved great success in reproducing the microstructures and properties of self-assembled polymer chains^73–78^. Recently, Chen et al. trained the traditional DNN to solve the MDE in diblock copolymer systems and the static Schrodinger equation in quantum systems, and the efficiency of the solver was analyzed^67,79^. In this work, we developed a PINN with residual units, which combines with the GLHS, to solve the forward and backward MDEs used in polymer physics.

Several important issues are addressed in the current study. In the following section, we describe the PINN with residual units and mixed sampling method. Then, we solve the forward and backward problems in MDEs as examples to examine the PINN solver based on the mixed data sampling by optimizing the parameters in PINN and mixed data samplings. We also compare the PINN to the numerical results and the traditional NN to verify the accuracy and efficiency of the PINN. The research summary is presented in final section.

## Neural network and data sampling

### PINN with residual units

**Figure 1:**
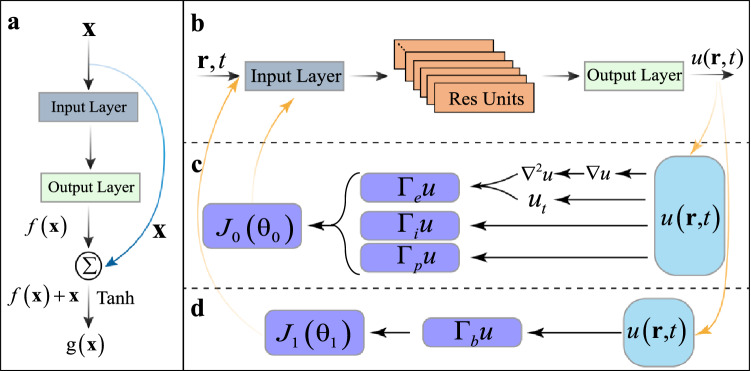
PINN with residual units. (**a**) The composition of residual unit; (**b**) PINN based on residual units; (**c**) The forward solution for the partial derivatives with physical constraints; (**d**) The backward solution for the partial derivatives with physical constraints.

We describe PINN with residual units, as shown in Fig. 1. To solve the complex problems in network convergence caused by gradient disappearance or network degradation in the traditional neural network^80,81^, we apply the neural network based on residual units to solve second-order PDEs. We describe a residual unit in Fig. 1a, where the input layer (IL) is a neural network layer with weight, biases, and an activation function. For this network layer, the output of tensor $\textbf{X}^{i-1}$ fed into the network is

1 $$\begin{aligned} {\textbf{I}}{\textbf{L}}(\textbf{X}^{i-1}) = \sigma (\textbf{W}^{i}\textbf{X}^{i-1} + \textbf{b}^{i}), \end{aligned}$$

where $\textbf{W}^{i} \in \mathbb {R}^{\textbf{n}_{i-1} \times \textbf{n}_i}$ is the weight parameter in the network layer, $\textbf{b}^{i} \in \mathbb {R}^{\textbf{n}_{i}}$ is the bias parameter in the network layer, and $\textbf{n}^{i}$ represents the network width of the current layer, that is, the number of neurons. The activation function $\sigma (\cdots )$, which is nonlinear, is the key factor in the universal approximation of the neural network. In general, the activation function selects nonlinear functions, such as sigmoid and tanh. Here, we choose tanh as the activation function^22,66^, i.e.,

2 $$\begin{aligned} \sigma (\textbf{X}) = Tanh(\textbf{X}) = \frac{e^{\textbf{X}} - e^{-\textbf{X}}}{e^{\textbf{X}} + e^{-\textbf{X}}}. \end{aligned}$$

The output layer (OL) is an ordinary network layer with weights and biases. For input tensor $\textbf{X}^{i-1}$, its output can be expressed as

3 $$\begin{aligned} {\textbf{O}}{\textbf{L}}(\textbf{X}^{i-1}) = \textbf{W}^{i}\textbf{X}^{i-1} + \textbf{b}^{i}. \end{aligned}$$

The residual unit is constructed by combining IL and OL, as illustrated in Fig. 1a. First, input tensor $\textbf{X}^{i-1}$ in the residual unit goes through IL and OL to obtain

4 $$\begin{aligned} f(\textbf{X}^{i-1}) = \textbf{W}^{i+1}\sigma (\textbf{W}^{i}\textbf{X}^{i-1}+\textbf{b}^{i})+\textbf{b}^{i+1}. \end{aligned}$$

Second, input tensor $\textbf{X}^{i-1}$ is connected by jump identity, and the output is obtained by the tanh activation function after accumulation with $f(\textbf{X}^{i-1})$, namely,

5 $$\begin{aligned} g(\textbf{X}^{i-1}) = \sigma (f(\textbf{X}^{i-1}) + \textbf{X}^{i-1}). \end{aligned}$$

This residual unit design enables us to transmit the input data directly from the lower network layer to the higher layer. This process differs from the common stacking of two neural network layers. Therefore, the residual unit can facilitate the optimization process of the neural network and solve the problem of network degradation to a certain extent.

Parameterized parabolic-type PDEs, such as MDE, can be expressed in a general form as follows:

6 $$\begin{aligned} u_t + N[u(\textbf{r}, t);\lambda ] = 0, \textbf{r} \in \Omega , t \in [0, T], \end{aligned}$$

where $u(\textbf{r}, t)$ denotes the solution with respect to the time and space variables, $N[u(\textbf{r}, t);\lambda ]$ represents the differential operator parameterized by $\lambda$, $\Omega$ is the spatial definition domain, and $u_t$ denotes the partial derivative with respect to time *t*.

In accordance with the universal approximation of neural networks^19,82^, the solution $u(\textbf{r}, t)$ in PDE can be equivalently expressed through DNN. In the current study, we use a PINN with residual units to solve PDE, as shown in Fig. 1b, instead of a common neural network. In such a PINN, the initial variables are inputted into the input layer and sent to several residual units (Fig. 1a), where the solutions are assessed by the physics-informed loss functions. Then, we obtain the solution $u(\textbf{r}, t)$ in the output layer.

We illustrate the calculations for the partial derivatives with physical constraints in the solution process in Fig. 1c and d, and the chain derivative rule based on automatic differentiation is used^35^. In the PINNs, the forward and backward solutions in the PDEs are learned by optimizing the loss functions with physical information. The forward solution process for the PDE in the neural networks is shown in Fig. 1c. The physical constraints of the differential equation can be defined as

7 $$\begin{aligned} \Gamma _e = u_t + N[u(\textbf{r}, t);\lambda ]. \end{aligned}$$

The representation network needs to learn that the judgment mode of the solution of the differential equation is $\Gamma _e \rightarrow 0$, which simultaneously satisfies the physical constraints with the periodic boundary condition (PBC) $\Gamma _p \rightarrow 0$, and the initial condition $\Gamma _i \rightarrow 0$. Then, we can define the total loss function as

8 $$\begin{aligned} J_0(\theta _0) = \Gamma _e + \Gamma _p + \Gamma _i, \end{aligned}$$

where $\theta _0$ denotes an intermediate variable that includes the parameters appearing in $\Gamma _e$, $\Gamma _p$, and $\Gamma _i$. The learning task stops in the forward process when $J_0(\theta _0) \rightarrow 0$. For the reverse problem, as shown in Fig. 1d, the network needs to satisfy $\Gamma _e \rightarrow 0$ and the constraints created by existing data in the network $\Gamma _b \rightarrow 0$, which will be described in detail in solving MDE section. In the backward process, the gradient of a certain underlying output can be expanded as

9 $$\begin{aligned} \frac{\partial {J_1}}{\partial {\textbf{X}^{i-1}}} = \frac{\partial {J_1}}{\partial {g}}\frac{\partial {g}}{\partial {\textbf{X}^{i-1}}} + \frac{\partial {J_1}}{\partial {g}}\frac{\partial {g}}{\partial {f}}\frac{\partial {f}}{\partial {\textbf{X}^{i-1}}}. \end{aligned}$$

Equation (9) shows that the gradient of the bottom output of the network can be decomposed into two terms. The first term indicates that the wrong signal can be directly transmitted to the bottom without any intermediate weight matrix transformation, thus alleviating the problem of gradient dispersion to a certain extent. The gradient will not disappear even if the weight of the intermediate layer matrix is small. The residual unit, which has been successfully applied to image recognition^32,34^, provides an efficient tool to solve the backward problems in PDEs in the current study.

### Mixed GS and LHS method

The basic problem in solving PDEs by using a neural network is to produce results that satisfy the physical conditions in the differential equations, where the data points in the defined domain are fed to the neural network. Thus, selecting appropriate data points in the training process of DNNs is crucial. In the current work, we adopt a mixed sampling method (GLHS), i.e., mixture of Cartesian GS and LHS, in the PINN solver.

**Figure 2:**
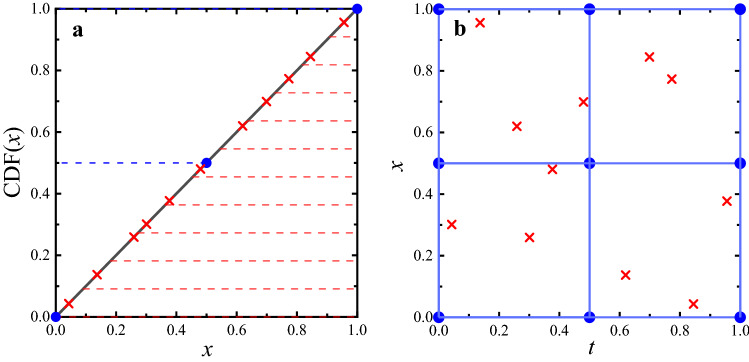
The data sampling distribution of GLHS. Take a total of 20 data, 2 dimensions, each dimension in the domain of [0,1] as an example. The blue dots represent the Cartesian grid data points and the crossed dots denote the data in LHS. (**a**) The data sampling on each dimension; (**b**) The overall data distribution after mixing.

**Figure 3:**
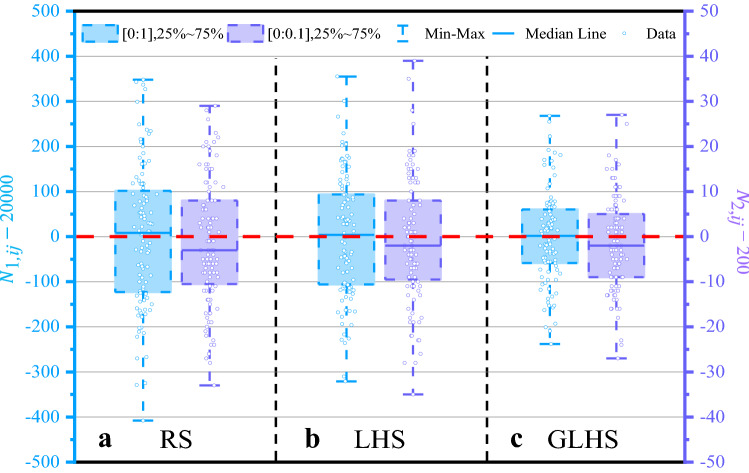
Comparison of three types of sampling. The blue Y-axis on the left of the figure represents the number of data $N_{1,ij}$ in 100 equal square data collections area of $\Omega _1=[0,1;0,1]$, and the purple Y-axis on the right represents the number of data $N_{2,ij}$ in 100 data collections area of $\Omega _2=[0,0.1;0,0.1]$. The box represents the data distribution within the range of 25%-75% of $N_{1,ij}$ and $N_{2,ij}$. The solid line in the box is the median line of data distribution, the line cap represents the maximum and minimum value of data distribution. The red dotted line represents the ideal number of data distributions, 20,000 and 200 respectively.

For the GLHS method, we assume that the number of data points in Cartesian GS is $\alpha {N}$, where *N* is the total number of data points and $\alpha$ is a proportionality coefficient, i.e., $\alpha \in [0,1]$. All the data are located in the domain $\mathbb {R}^{\Omega }$. We allocate the $\alpha {N}$ data points to the grid points where the *n*-dimensional Cartesian grids are equally divided into INT($\root n \of {\alpha {N}}$) grids in one dimension. INT($\cdots$) denotes the integer part of the number. This condition means that a total of $N_G$ = [INT$(\root n \of {\alpha {N}})]^{n}$ data points are sent to the grid in an *n*-dimensional space, where one data point corresponds to one grid point. Then, the remaining data points, $N_L$ are sampled by the LHS method, where $N_L = N - [$INT($\root n \of {\alpha {N}})]^{n}$. In LHS, the $N_L$ data points are sent to $N_L$ equal subdomains for RS, where each subdomain corresponds to one LHS data point^62,64^. To effectively describe the GLHS method, we show a simple example in Fig. 2, where $\alpha =0.5$, $N=20$, $n=2$. For simplicity, we use the definition domain $\Omega _1=[0, 1;0, 1]$ as an example (other examples are given in Fig. S1 of Supplementary information). The blue dots represent the Cartesian grid data points, and the crossed dots denote the data points in LHS. Clearly, 3 data points exist in the Cartesian grid sampling, and 11 data points are present in LHS, where the domain is divided into 11 subdomains in one dimension and each data point in a subdomain is randomly sampled, as shown in Fig. 2a. We expect that the extracted random data are evenly distributed in the definition domain, so a linear cumulative density function, CDF$(x)=\Omega ^{-1}{x}$, is used in each dimension for LHS. Then, the total data points in two dimensions are calculated by the Cartesian product, i.e., total of 9 data points in the Cartesian grids. Meanwhile, 11 data points are randomly paired into two dimensions via a cumulative linear density function, $\Omega _1=[0, 1;0, 1]$, as shown in Fig. 2b. Finally, the Cartesian grid and LHS data are randomly mixed to obtain GLHS data as the final input data for PINN.

We present an example to illustrate the advantages of the GLHS method in Fig. 3, where three types of sampling, namely, RS, LHS, and GLHS, are compared. In each sampling type, two types of two-dimensional definition domains, i.e., $\Omega _1=[0, 1;0, 1]$ and $\Omega _2=[0, 0.1;0, 0.1]$, are used as examples. A total of 2,000,000 and 20,000 data points are randomly imported into the two definition domains. To examine the uniformity of the data distribution in $\Omega _1$ and $\Omega _2$, we divide the two definition domains into 100 equal square data collection areas and labeled the number of data points in each data collection area as $N_{1,ij}$ and $N_{2,ij}$ over all the regions in $\Omega _1$ and $\Omega _2$. The subscripts *i* and *j* denote the *ij*-th collection area in the two dimensions. We plot more detailed data distributions in the two-dimensional definition domains in Fig. S2 of Supplementary information. Thus, 100 data collection areas are distributed in each definition domain for RS, LHS, and GLHS. We sort $N_{1,ij}$ and $N_{2,ij}$ into a sequence on the basis of their values and label the $N_{1,ij}$ and $N_{2,ij}$ in the middle 25–75% of the sequence as a square box with dotted lines, as shown in Fig. 3a–c. Among the three sampling types, RS has the largest box and GLHS has the smallest one in the $\Omega _1$ domains, indicating that GLHS possesses the most uniform data distributions in the definition domain.For the $\Omega _2$ domains, GLHS also has the most uniform data distribution, but the data points at the middle of the sequence have values below 200, which is the average number of data points for the collection data in $\Omega _2$ domains. This result may due to the reason that the amount of input data is not large enough in the $\Omega _2$ domains. Recently, simple LHS with point transformation was used to increase the uniformity of data distribution^83^. In this study, we observe that GLHS has an obvious advantage over RS and LHS, although RS and LHS have similar sampling procedures in the uncertainty analysis^62^.

## Solving for forward and backward MDE

In polymer physics, MDE is a key equation in self-consistent field theory and has been numerically solved by many methods^70,71^. We adopt MDE as an example to illustrate the use of PINN based on GLHS in solving forward and backward problems. First, we present the general form of forward and backward MDE. Second, we discuss the efficiency of GLHS and PBC loss functions in solving for MDE. Lastly, we discuss the forward and backward problems in MDE solutions by using PINN based on GLHS.

### Problem setup

As an example, we take a simple form of forward and backward MDE with the initial conditions and PBCs, which can be expressed as

10 $$\begin{aligned} \frac{\partial }{\partial {t}}u(x, t) = \frac{\partial ^{2}}{\partial {x}^{2}}u(x, t) - \lambda \sin (2\pi {x})u(x,t), \end{aligned}$$

where the initial condition is $u(x,0) = 1$ and the periodic boundary condition is $u(0,t) = u(L,t)$. Here, only two dimensions are used; *L* is the period in the *x* dimension. And when $\lambda$ is a given parameter, such as $\lambda = 7$ used in this work, the problem becomes a forward problem. When $\lambda$ is an unknown parameter, solving the equation becomes a backward problem. MDE is a linear, second-order, parabolic-type PDE in which the forward problem is addressed by DNN^28,79^. However, the backward problem in MDE still needs to be understood. Here, we use PINN based on GLHS to solve the forward and backward problems in MDE.

### Optimization scheme for GLHS and PINN

The neural network and sampling method should be optimized when used to solve a special PDE. In this study, the core issue in GLHS is how to find the optimizing mixture coefficient $\alpha$ as the special PDE; meanwhile, depth *D* and width *W* in the neural network are also important parameters in PINN and should be optimized. Given that the two types of parameters are independent, we adopt the independent variable method to optimize the GLHS and PINN parameters.

**Figure 4:**
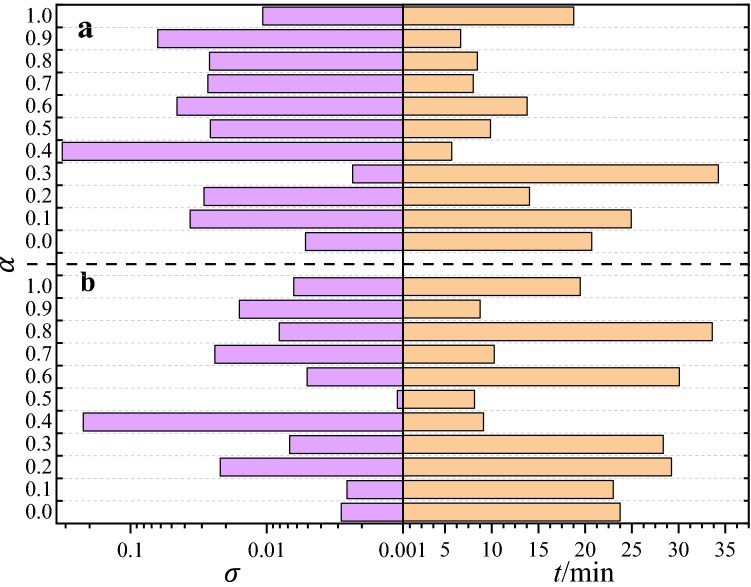
Comparison of training time (*t*) and standard error ($\sigma$) with two data sampling methods in the PINN with residual units. (**a**) The mixture of GS and RS at $\alpha :(1-\alpha )$ ratio, where $\alpha =0$ denotes simple random sampling; (**b**) The mixture of GS and LHS, i.e., GLHS, at $\alpha :(1-\alpha )$ ratio, where $\alpha =0$ denotes simple LHS sampling. The purple bars on the left represent the standard error of network training results, and the abscissa (0.001–0.5) is amplified by log$_{10}$. The yellow bars on the right represent the time spent on network training.

The primary issue in GLHS, which is how to find optimizing mixture coefficient $\alpha$ as the special PDE, is solved in PINN. We further describe the GLHS by comparing it with other mixture sampling methods in solving MDE using PINN, as shown in Fig. 4. For a comparison, we adopt two types of mixture samplings, namely, the mixture of random sampling and grid sampling, as shown in Fig. 4a, and GLHS in Fig. 4b, respectively. The data used are from solving for MDE in Eq. (10) with PBC of $L=1.0$, where the number of residual units is 6 and the width of neural network layer is 20 in PINN. Then, we use a total of $N=301 \times 301$ data points in the mixture of random and grid samplings as well as GLHS, where the number of data points in Cartesian GS is $\alpha {N}$. To explain the efficiency of the sampling method, we employ sampling standard errors as follows:

11 $$\begin{aligned} \sigma = \root \of {\sum _{i=1}^{N}\frac{(u_i-u_{0i})^2}{N-1}}, \end{aligned}$$

where $u_i$ is the PINN solving value and $u_{0i}$ is the numerical value solved by the Crank-Nicholson method, which has been used for MDE in previous simulation calculations^74,84–86^. The sum takes over all the discrete data points in the definition domains used in the Crank-Nicholson method, and *N* is the total number of discrete data points.

In the mixture of random and grid sampling case, the results indicate that it is difficult to optimize $\sigma$ and *t* as $\alpha$ varies; *t* is the corresponding training time, as shown in Fig. 4a. In particular, when standard error $\sigma$ has the minimum value, training time *t* is the longest. In the GLHS case, $\sigma$ can reach its minimum value and the *t* minimum value simultaneously, as shown in Fig. 4b. We optimize GLHS with $\alpha =0.5$ which indicates that the minimum error and training time can be achieved when the data numbers in GS are equal to those in LHS. Figure 4 also show the results of RS ($\alpha =0.0$ in Fig. 4a), LHS ($\alpha =0.0$ in Fig 4b) and GS ($\alpha =1.0$ in Fig. 4a and b) by using PINN. We can find that in terms of *t* and $\sigma$, these three sampling methods are not good choices for data samplings, comparing to the GLHS with $\alpha =0.5$. These results agree with those data distributions described in Fig. 3. We note that the LHS has been used in PINN^28^. Here, we try to use the GLHS instead of LHS to enhance the efficiency for solving PDEs in PINN.

PBC is important in PDE, especially when handling a bulk polymer system. The boundary condition has been strengthened in previous studies when designing the structure of neural networks^36,87,88^. Thus, we consider PBC optimization when choosing the proper network parameters. As illustrated in Eq. (10), PBC in spatial period *L* can be regarded as $u(0,t)=u(L,t)$. The PBC in numerical methods can be done by setting the calculation cell size to *L*, which satisfies $u(0,t)=u(L,t)$^72,74^. However, the PBC in neural networks has to satisfy the left condition $u(x,t)=u(x-L,t)$, the right condition $u(x,t)=u(x+L,t)$, or both, that is, the squeeze period condition where $x \in [0,L]$. To identify the optimized PBC among the three PBCs, we construct the following types of loss functions.

12 $$\begin{aligned} J_{p1}= & {} \frac{1}{N}\sum _{i=1}^{N}[u(0,t_i)-u(L,t_i)]^2, \end{aligned}$$

13 $$\begin{aligned} J_{p2}= & {} \frac{1}{N}\sum _{i=1}^{N}[u(x_i,t_i)-u(x_i-L,t_i)]^2, \end{aligned}$$

14 $$\begin{aligned} J_{p3}= & {} \frac{1}{N}\sum _{i=1}^{N}[u(x_i,t_i)-u(x_i+L,t_i)]^2, \end{aligned}$$

15 $$\begin{aligned} J_{p4}= & {} J_{p2}+J_{p3}, \end{aligned}$$

where $x_i \in [0,L]$ and $t_i \in [0,T]$. Summation is performed over the two domains. Equation (12) is commonly used in numerical methods and listed here for comparison.

**Figure 5:**
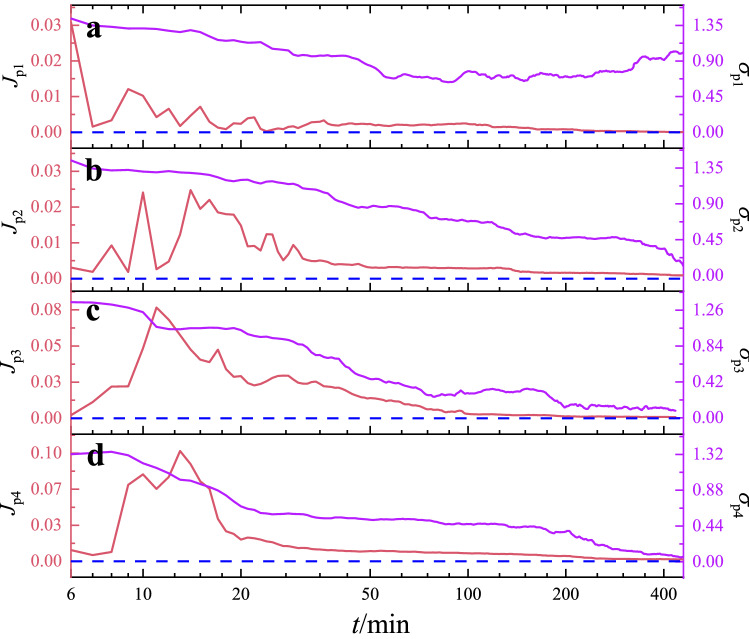
Comparison of training processes for four types of PBC loss functions. The red line and the red Y-axis on the left represent the change of the loss functions, while the purple line and the purple Y-axis on the right represent the standard error between the network results and analytical solutions.

The training processes for the four PBC loss functions are shown in Fig. 5. The standard errors $(\sigma _p)$ from the results of PINN, i.e., *u*(*x*, *t*), and from the results of the Crank-Nicholson numerical method, i.e., $u_0{(x,t)}$ are also listed on the right side. In Type 1, as shown in Fig. 5a, although loss function $J_{p1}$ converges to a desired value, standard error $\sigma _{p1}$ is too large to achieve correct solutions. For Types 2 and 3, as shown in Fig. 5b and c, loss functions $J_{p2}$ and $J_{p3}$ can converge to the desired values, and standard errors $\sigma _{p2}$ and $\sigma _{p3}$ are still able to converge to the desired values. However, when we use the squeeze period condition, loss function $J_{p4}$ and standard error $\sigma _{p4}$ can converge to the desired value in the training processes. Furthermore, we calculate the Pearson correlation coefficient, $\rho _{X,Y}$, between standard error $\sigma _p$ and loss function $J_p$, which can be generally defined as

16 $$\begin{aligned} \rho _{X,Y}=\frac{cov(X,Y)}{\sigma _X\sigma _Y}, \end{aligned}$$

where *X* and *Y* denote standard error $\sigma _p$ and loss function $J_p$, respectively. $\sigma _X$ denotes the standard error of *X*, and *cov*(*X*, *Y*) represents the covariance between *X* and *Y*. Then, we list the $\rho _{X,Y}$ for the four types of PBC in Table 1. The data indicate that the first three types of PBC have a strongly correlation between $\sigma _p$ and $J_p$, but $\sigma _p$ and $J_p$ have almost no correlations in the squeeze boundary condition. This result indicates that the squeeze period method is feasible and can effectively improve the training accuracy of solving PDEs with PBC in PINN.

**Table 1:** Pearson correlation coefficient between standard error $\sigma _L$ and loss function $J_L$.

PB	$\rho _{X,Y}$
Numerical	0.72590
Left PB	0.85306
Right PB	0.70024
Squeeze PB	0.02463

Then, we optimize the neural network parameters, namely, the depth and width in PINN(*D* and *W*). *D* is the number of residual units, and *W* is the number of neural units in the layer. *D*, *W*, and the amount of trained data(*N*) have a great influence on the output accuracy of the neural network. We train PINN in a multi-parameter space of *D*, *W*, and *N*, where $D \in [3,4,5,6,7,8]$, $W \in [10,15,20,25,30]$, and $N \in [2000,4000,6000,8000,10000,20000]$, as indicated in Table 2, where only several typical combinations are shown. Indeed, $D \times W \times N=180$ combinations exist in the full parameter space of [*D*, *W*, *N*]. Other detailed combinations can be found in Figs. S3 and S4 of Supplementary information.

**Table 2:** Standard error ($\sigma$) and loss function (*J*) corresponding to different parameters, depth (*D*), width (*W*), and number of data (*N*).

*D*	*W*	*N*	*J*	$\sigma$
6	20	20000	$5.23\times 10^{-5}$	$1.1\times 10^{-3}$
4	25	6000	$9.84\times 10^{-5}$	$1.2\times 10^{-3}$
3	30	8000	$9.40\times 10^{-5}$	$1.3\times 10^{-3}$
3	30	6000	$1.10\times 10^{-4}$	$1.4\times 10^{-3}$
4	25	20000	$7.30\times 10^{-5}$	$1.5\times 10^{-3}$
3	15	10000	$3.88\times 10^{-5}$	$2.0\times 10^{-3}$
8	20	6000	$7.39\times 10^{-5}$	$2.4\times 10^{-3}$
3	25	10000	$1.67\times 10^{-4}$	$2.9\times 10^{-3}$
5	25	4000	$2.44\times 10^{-4}$	$3.2\times 10^{-3}$
6	30	2000	$1.96\times 10^{-4}$	$3.5\times 10^{-3}$

**Figure 6:**
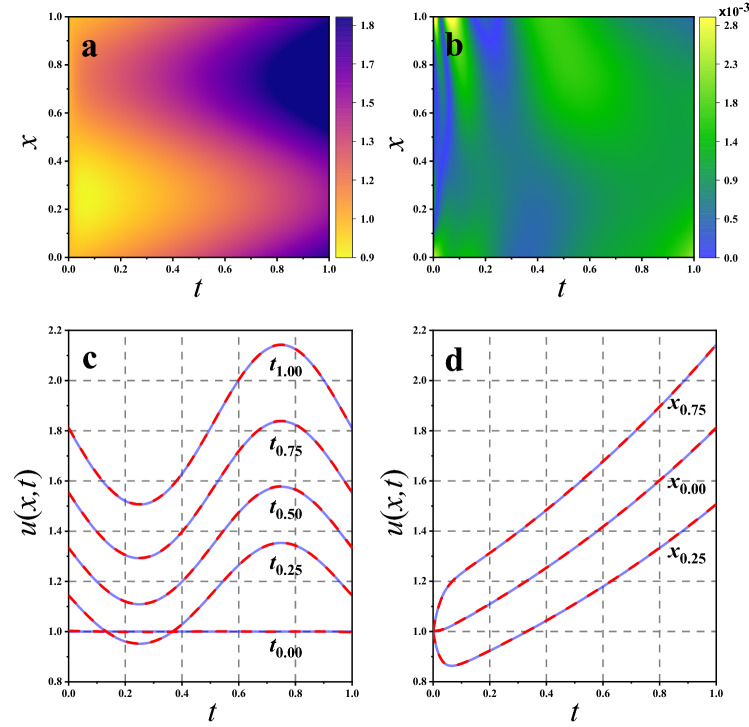
Comparison of PINN results with numerical results. (**a**) The overall view in the two dimensional space of PINN results *u*(*x*, *t*); (**b**) Diagram of the difference between PINN results and numerical results; (**c**) The accuracy in one-dimensional space with given $t={0.00,0.25,0.50,0.75,1.00}$; (**d**) The accuracy in one-dimensional space with given $x={0.00,0.25,0.75}$. The blue dotted line represents the numerical results and the red line represents PINN results.

We use loss functions *J* and standard errors $\sigma$ to screen the desired parameters. The optimal parameter corresponds to the minimum $\sigma$, which is the combination with $D=6$, $W=20$, and $N=20000$. Generally, an increase in *N* leads to a decrease in *J* and $\sigma$, indicating a simple relationship. However, the optimal output relies on the complex combination of *D* and *W*. The data in Table 2 reveal that the best optimization combination is the parameter combination of $D=6$, $W=20$, and $N=20000$. Generally, using a number of hidden layers during training results in a large precision loss, but the optimization combination here shows that this is not the case^89^.

### Forward and backward solutions

First, we discuss the forward solutions for MDE by PINN. We use the optimal PINN parameters, $D=6$, $W=20$ and $N=20000$, and the GLHS parameter, $\alpha =0.5$, to solve the forward and backward problems in MDEs appearing in Eq. (10). We take the definition domains $x \in [0,1]$ and $t \in [0,1]$, and to evaluate the accuracy of the PINN solution *u*, we define the relative errors with respect to the numerical solutions $u_0$ as follows:

17 $$\begin{aligned} \delta _{ij}=\vert \frac{u(x_i,t_j)-u_0{(x_i,t_j)}}{u_0{(x_i,t_j)}}\vert , \end{aligned}$$

where subscripts $x_i$ and $t_j$ correspond to definition domains *x* and *t*,respectively. We plot the neural network results and compare them with the numerical results in Fig. 6. We adopt an overall view in the two-dimensional space, as shown in Fig. 6a and b. The results indicate that the PINN results have high accuracy within $10^{-3}$ distributed in the defintion domain. Then, we examine the accuracy in one-dimensional space with given *x* or *t*, as shown in Fig. 6c and d, respectively. The data confirm that our PINN results vary with the given *x* or *t*. Furthermore, we illustrate the data for the given *x* or *t* in Table 3, where the relative errors are also listed.

**Table 3:** The relative errors corresponding to different time and space positions.

*t*	$\delta$	*x*	$\delta$
0.00	$8.55\times 10^{-4}$	0.00	$8.39\times 10^{-4}$
0.25	$4.95\times 10^{-4}$	0.25	$7.32\times 10^{-4}$
0.50	$8.43\times 10^{-4}$	0.50	$6.83\times 10^{-4}$
0.75	$7.31\times 10^{-4}$	0.75	$7.03\times 10^{-4}$
1.00	$6.57\times 10^{-4}$	1.00	$8.90\times 10^{-4}$

For the inverse problem in MDE, discovering the unknown parameters is difficult due to the complex physical constraints and gradient disappearance. Unlike in the case where a sparse regression method is employed to determine PDE by time series measurements in the spatial domain^21^, in this study, we design an interleaved training method with discontinuous double-loss functions $\Gamma _e$ and $\Gamma _b$. Loss function $\Gamma _e$ is defined in Eq. 7, and $\Gamma _b$ can be defined as

18 $$\begin{aligned} \Gamma _b=\frac{1}{N}\sum _{i=1}^{N}[u(x_i,t_i)-u_0{(x_i,t_i)}]^2. \end{aligned}$$

The sum over all the definition domains, $u_0{(x_i,t_i)}$,is the numerical solution and taken as the standard value. In this method, we search for unknown parameter $\lambda$ through loss function $\Gamma _e$ and optimize the network solution through $\Gamma _b$. That is, $\Gamma _e$ is optimized to screen parameter $\lambda$ at the first training stage. Then, we lock up the parameter $\lambda$ to optimize the network solution $\Gamma _b$ and obtain a high-accuracy network solution.

**Figure 7:**
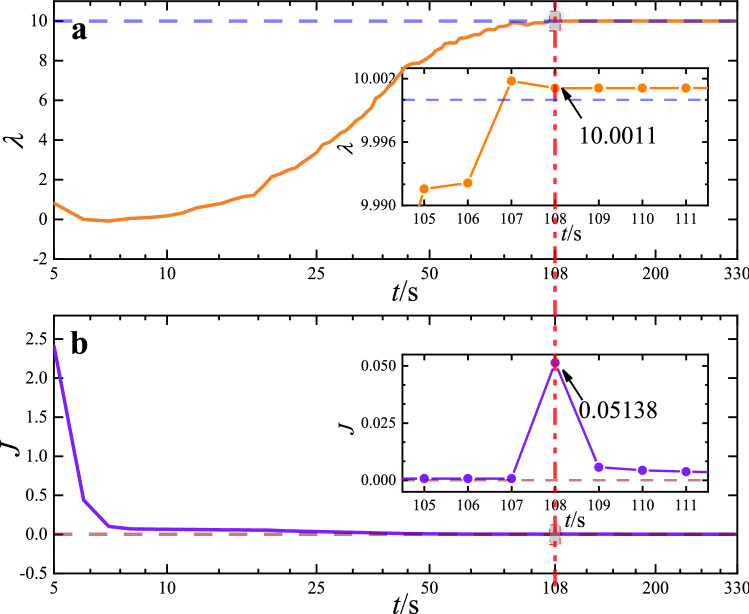
The training process of solving unknown parameters of inverse problem in MDE. (**a**) The optimization process of unknown parameter $\lambda$; (**b**) The optimization process of loss function *J*. The blue and brown dotted lines represent $\lambda =10$ and $J=0$, respectively.

Specifically, we construct the PINN with four residual units, each of which has a full-connection layer width of 20 neural units, to solve the inverse problems. We optimize the network parameters and unknown parameters $\theta _0{(w,b,\lambda )}$ via the loss function $J_0{(\theta _0)}$ at the first stage. Then, we lock up parameter $\lambda$ and optimize $\theta _1{(w,b)}$ by using loss function $J_1{(\theta _1)}$ at the second stage until the loss function reaches $10^{-5}$, as shown in Fig. 7. We label the blue and brown dotted lines as $\lambda =10$ and $J_0{(\theta _0)}=0$, respectively. The results indicate that parameter $\lambda =10.00111$ at $t=108s$ when $J_0{(\theta _0)}\le 10^{-5}$, as shown in Fig. 7a. At this point, we lock up parameter $\lambda$, and loss function $J_0{(\theta _0)}$ is automatically awitched to $J_1{(\theta _1)}$. Then, network parameter $\theta _1{(w,b)}$, which determines the network solution, is further optimized at the second stage. We observe that the loss function is discontinuous at this point, as shown in the inserted part in Fig. 7b. Generally, the loss function exhibits a sudden drop when the optimizer switches in the training process^52^. We also observe a discontinuous loss function in a small region of $\Delta {J}=0.05$ when the loss function switches. In the current study, we obtain parameter $\lambda$ with high accuracy. The absolute error of parameter $\lambda$ is 0.0011, the relative error is $\delta _\lambda =0.011\%$, and the standard error is $\sigma _\lambda =0.0046$. In this work, we present a high-efficiency interleaved training method to search for the unknown parameters in the reverse problem in MDE, and it can be reasonably extended to other PDEs.

### Comparison between PINN and NN

**Figure 8:**
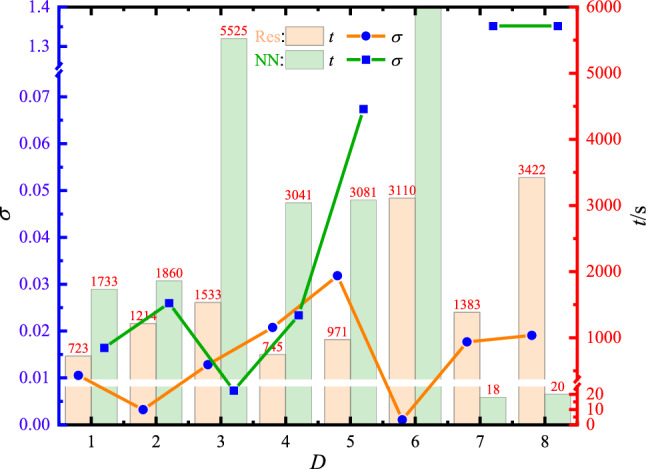
Comparison of training time (*t*) and standard error ($\sigma$) between PINN with residual units and traditional NN, Here, the width of neural network *W* is 20, data sampling number *N* is 20000, and the mixture coefficient of GLHS $\alpha$ is 0.5 in both cases. The yellow and green bars denote the training time *t* in the PINN and traditional NN cases, respectively, while the blue dots in the bars represent the corresponding standard error $\sigma$.

To illustrate the advantages of PINN with residual units, we compare the results from the PINN to the traditional NN by the standard error $\sigma$ and training time *t*, as shown in Fig. 8. Here, the traditional NN is constructed without the residual units, which is similar to the previous works^22,25^. For convenient comparison, the other conditions are set to be the same as in both cases, including the data samplings (GLHS with $\alpha$=0.5), the input data number( $N=20000$), and the width of network ($W=20$). Since the residual unit consists of two neural network layers, we take two network layers in traditional NN as a layer for more convenient comparison, so that there are 2*D* network layers in the traditional NN. Then, we only change the depth of PINN *D* to output the training times and standard errors in both cases. The results indicate that with the increase of the number of neural network layers, the advantages of PINN with residual units become more and more obvious. In particular, when *D* = 6, the traditional neural network encounters a gradient explosion, leading to the meaningless output of loss and the other parameters, as shown in Fig. 8; when *D* = 7 and 8, the traditional NN can hardly be optimized bacause of the problem of gradient disapperance. This problem results in the extremely short training times *t* and large standard errors $\sigma$. In addition, even in the shallow networks with small *D*, the training time of PINN with residual units is still superiority to those of traditional NN, as shown in Fig. 8. Furthermore, we have compared the efficiencies between the PINN and traditional NN with the other *W*, and the similar results were obtained, please see Tabel S1 in Supplementary information. We expect this PINN with residual untis based on the mixed data sampling to be applied to other works, especially to the three-dimensional PDEs, in the future.

## Summary

We developed a PINN based on GLHS to solve the forward and backward MDEs by optimizing the corresponding parameters. This solver provides high efficiency and accuracy in solving forward and backward problems in one-dimensional MDEs, and it effectively avoids the problems of gradient disappearance and network degradation existing in traditional feedforward neural networks. For the neural network, we properly designed residual units for PINN to solve MDE, and squeeze PBC was considered. For data sampling, we considered the mixture of GS and LHS. We believe that this method can also used in other dimensional, and we will confirm the view in the future.

Then, we optimized the parameters used in PINN by considering the loss function of PBC. Specifically, the depth and width of the neural network, *D* and *W*, were optimized. The results indicated that the squeeze condition is suitable for MDE. We also optimized GLHS data sampling by adjusting the mixture coefficient $\alpha$. The results revealed that the parameter combination $[D,W,N,\alpha ]$ should be optimized to [6, 20, 20000, 0.5] in the given MDE with high precision. We demonstrated how the hybrid solver deals with forward and backward problems in a special MDE. We compared the neural network solvers results to the numerical solutions and found good agreement. For the forward MDE, we obtained high-accuracy PINN solutions within $10^{-3}$ by analyzing the errors between the PINN solutions and the numerical results. For the inverse problem in MDE, we designed the ITM method to screen the unknown parameters. Unknown parameter $\lambda$ was locked up with relative error $\delta _\lambda =0.011\%$ and standard error $\sigma _\lambda =0.0046$. This PINN with residual units based on mixed data sampling GLHS can be generalized to other cases for other PDEs.

## Supplementary Information

Supplementary Information.

## Data Availability

The datasets used and/or analysed during the current study available from the corresponding author on reasonable request.
