# Supplementary material for: A physics-informed neural network based on mixed data sampling for solving modified diffusion equations
Source: Sci Rep. 2023 Feb 13;13:2491. doi: 10.1038/s41598-023-29822-3 (PMC9925766; doi:10.1038/s41598-023-29822-3)
Supplement: Supplementary file 1 — Supplementary Information. [file 41598_2023_29822_MOESM1_ESM.pdf]

# Supplementary information
# A hybrid neural network and data sampling solver for forward and backward modified diffusion equations

Qian Fang[1], Xuankang Mou[1], and Shiben Li[1,*]
[1] *Department of Physics, Wenzhou University, Wenzhou, Zhejiang 325035, China and*
[*] *Corresponding author: Shiben Li (E-mail: shibenli@wzu.edu.cn)*

## DATA SAMPLING BY GLHS METHOD

We show the example in Fig. S1, where epochs=$[0, 1000]$, $N = 2000$, $n = 2$, definition domain=$[0, 1; 0, 1]$. Fig. S1(a) shows the data sampling method in each dimension. The linear cumulative density function CDF($x$)=$\Omega_{-1}x$ is used in each dimension for LHS. The mixing ratio of Cartesian grid sampling and Latin hypercube sanpling varies with epochs. When epochs=0, the sampling method is Cartesian grid sampling; when epochs=1000, the sampling method is Latin hypercube sampling. Due to the large amount of data, we select the data in $\Omega_2 = [0, 0.1; 0, 0.1]$ to show the data distribution in different proportions, as shown in Fig. S1(b), (c), (d).

Fig. S2 shows the data distribution generated respectively by RS, LHS, and GLHS. We sample 2000000 data in $\Omega_1 = [0, 1; 0, 1]$.

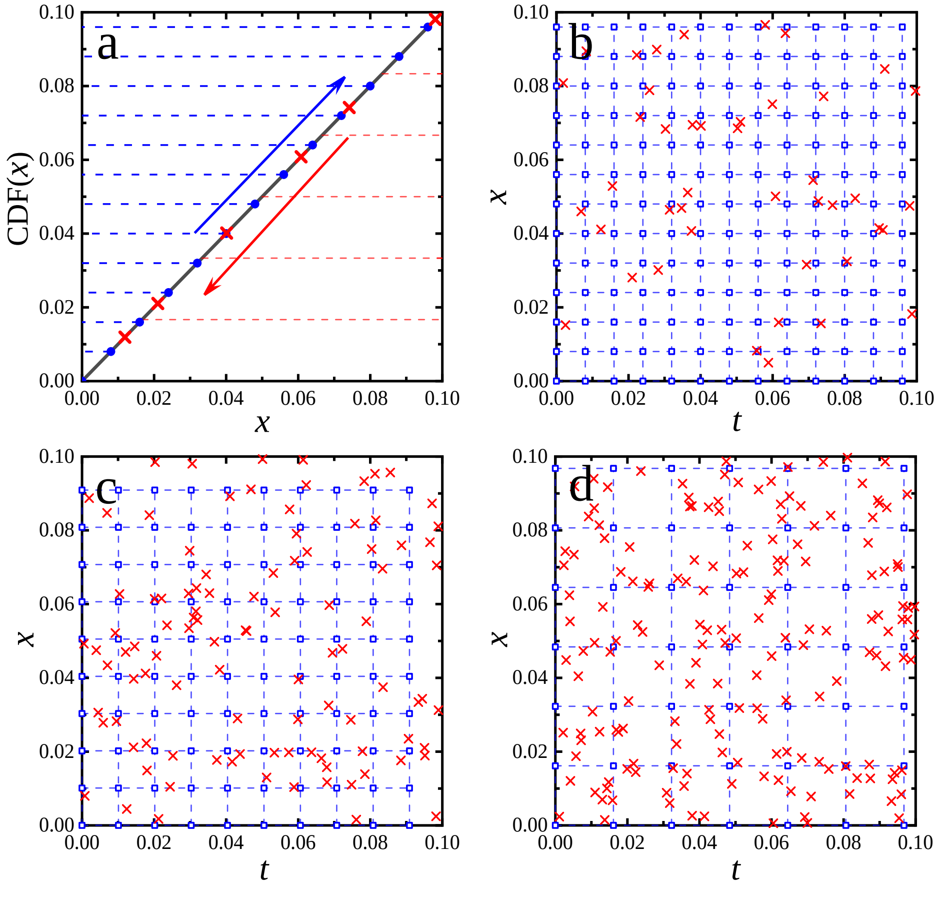


FIG. S1. The data sampling by GLHS method. The mixing ratio of GLHS varies with epochs=$[0, 1000]$. Data sampling number $N = 2000$ in 2-dimensional $\Omega_1 = [0, 1; 0, 1]$. We selected the data in $\Omega_2 = [0, 0.1, 0, 0.1]$ to show the change of data sampling. (a)Data sampling method in each dimension. The blue arrow shows that with the increase of training epochs, Cartesian grid method's layering points decrease bacause of the decrease of proportion. And the red arrow shows that Latin hypercube sampling method's laying points increase with the increase of training epochs; (b) When epochs is 200, Latin hypercube sampling data accounted for 20%; (c) When epochs is 500, Latin hypercube sampling data accounted for 50%; (d) When epochs is 800, Latin hypercube sampling data accounted for 80%.

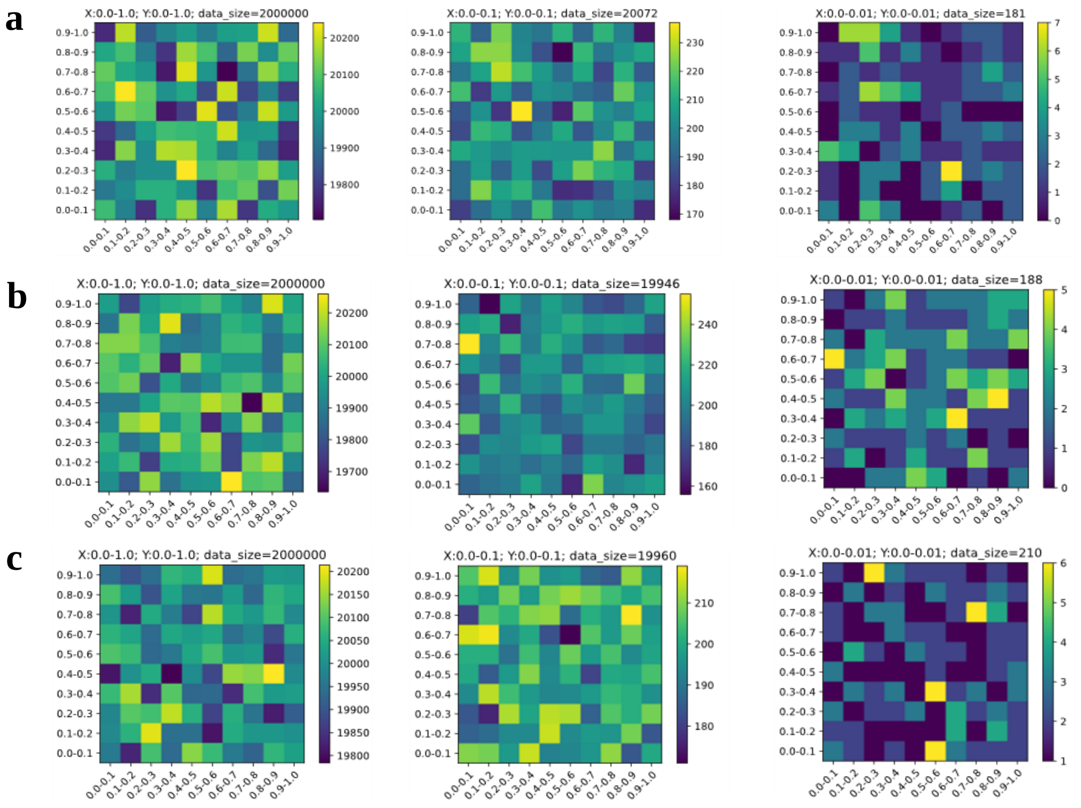


FIG. S2. Data distribution of three types of sampling. Data distribution of RS in $\Omega_1 = [0,1;0,1]$, $\Omega_2 = [0,0.1;0,0.1]$, $\Omega_3 = [0,0.01;0,0.01]$; (b) Data distribution of LHS in $\Omega_1 = [0,1;0,1]$, $\Omega_2 = [0,0.1;0,0.1]$, $\Omega_3 = [0,0.01;0,0.01]$; (c) Data distribution of RS in $\Omega_1 = [0,1;0,1]$, $\Omega_2 = [0,0.1;0,0.1]$, $\Omega_3 = [0,0.01;0,0.01]$;

## OPTIMIZATION FOR DEPTH AND WIDTH OF PINN AND TRAINED DATA

We train PINN in a multi-parameter space of $D \in [3,4,5,6,7,8]$, $W \in [10,15,20,25,30]$, and $N \in [2000,4000,6000,8000,10000,20$[illegible] Several typical combinations listed in Table 2 are also shown in Fig. S3. And the full detailed combinations are shown in Fig. S4.

## COMPARISN OF PINN WITH RESIDUAL UNITS AND TRADITIONAL NN

We compare the training time and standard error between PINN with residual units and traditional NN, where $D \in [3,4,5,6,7,8]$, $W \in [10,15,20,25,30]$, $N = 20000$. The results are listrd in Table S1.

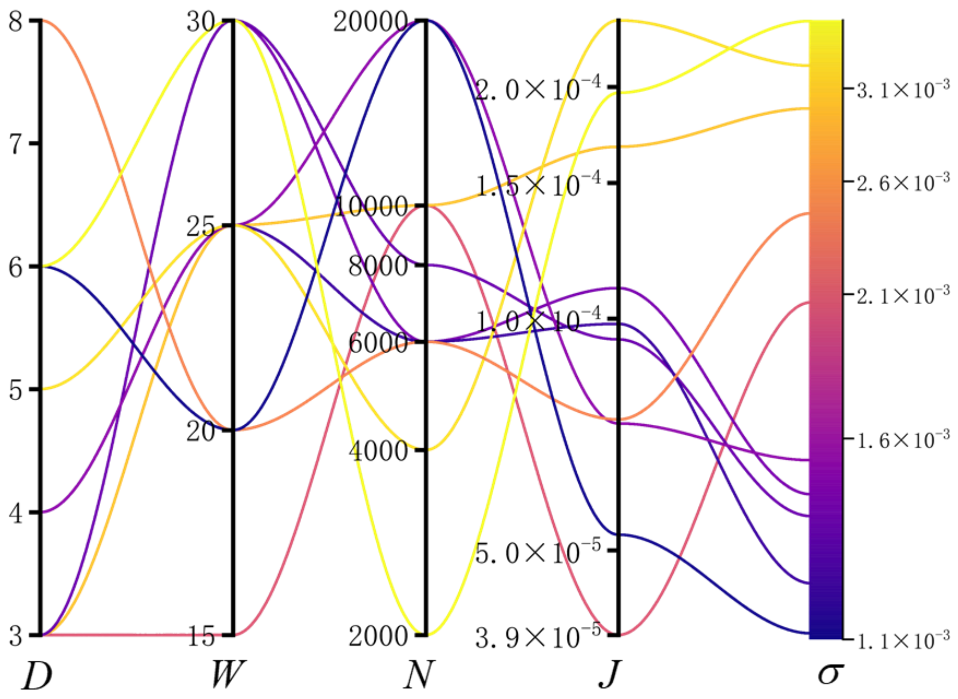


FIG. S3. Ten typical combinations of standard error corresponding to different parameters (depth ($D$), width ($W$), number of trained data ($N$), stanard error($\sigma$))

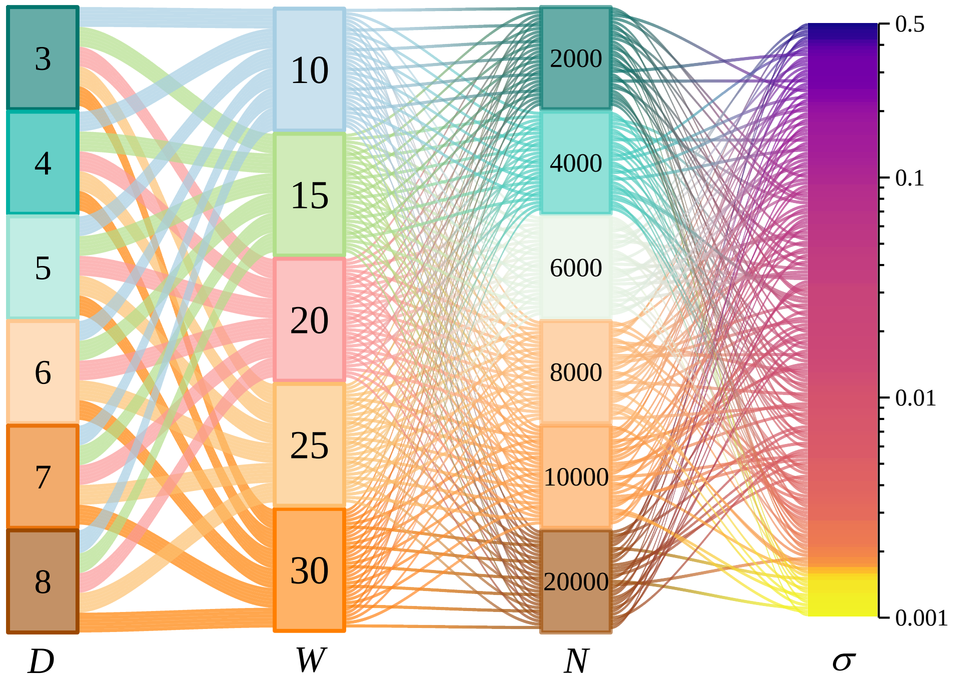


FIG. S4. Standard error corresponding to different parameters (depth ($D$), width ($W$), number of trained data ($N$), stanard error($\sigma$)) in the full parameter space of $[D, W, N]$.

TABLE S1. Comparison of training time($t$) and standard error($\sigma$) between PINN with residual units and traditional NN.

| $N = 20000$ | | PINN with residual units | | Traditional NN | |
|---|---|---|---|---|---|
| 3 | 10 | $2.20 \times 10^{-2}$ | 773 | $4.01 \times 10^{-1}$ | 3910 |
| | 15 | $3.17 \times 10^{-2}$ | 837 | $8.40 \times 10^{-3}$ | 5690 |
| | 20 | $1.28 \times 10^{-2}$ | 1533 | $7.30 \times 10^{-3}$ | 5525 |
| | 25 | $7.47 \times 10^{-3}$ | 2111 | $1.87 \times 10^{-2}$ | 3409 |
| | 30 | $4.05 \times 10^{-3}$ | 2228 | $1.10 \times 10^{-3}$ | 18566 |
| 4 | 10 | $3.37 \times 10^{-1}$ | 1317 | $5.20 \times 10^{-3}$ | 12762 |
| | 15 | $5.07 \times 10^{-3}$ | 2138 | $4.54 \times 10^{-2}$ | 2294 |
| | 20 | $2.08 \times 10^{-2}$ | 745 | $2.34 \times 10^{-2}$ | 3041 |
| | 25 | $1.49 \times 10^{-3}$ | 3187 | $4.02 \times 10^{-1}$ | 12208 |
| | 30 | $9.07 \times 10^{-3}$ | 2228 | $4.02 \times 10^{-1}$ | 17737 |
| 5 | 10 | $3.93 \times 10^{-2}$ | 576 | $4.52 \times 10^{-1}$ | 1907 |
| | 15 | $3.44 \times 10^{-2}$ | 499 | $4.22 \times 10^{-2}$ | 4862 |
| | 20 | $3.18 \times 10^{-2}$ | 971 | $6.74 \times 10^{-2}$ | 3081 |
| | 25 | $3.80 \times 10^{-2}$ | 1232 | $2.07 \times 10^{-2}$ | 3426 |
| | 30 | $1.30 \times 10^{-2}$ | 1398 | $1.38 \times 10^{-2}$ | 8651 |
| 6 | 10 | $4.48 \times 10^{-1}$ | 1148 | $5.69 \times 10^{-2}$ | 2683 |
| | 15 | $9.75 \times 10^{-2}$ | 1835 | NaN | 20301 |
| | 20 | $1.06 \times 10^{-3}$ | 3110 | NaN | 20597 |
| | 25 | $1.09 \times 10^{-2}$ | 3083 | $2.09 \times 10^{-2}$ | 6871 |
| | 30 | $8.04 \times 10^{-3}$ | 3304 | $9.20 \times 10^{-3}$ | 16230 |
| 7 | 10 | $2.35 \times 10^{-2}$ | 888 | 1.35 | 19 |
| | 15 | $2.05 \times 10^{-2}$ | 2410 | 1.35 | 16 |
| | 20 | $1.77 \times 10^{-2}$ | 1383 | 1.35 | 18 |
| | 25 | $2.25 \times 10^{-2}$ | 1906 | $4.18 \times 10^{-2}$ | 5135 |
| | 30 | $8.41 \times 10^{-3}$ | 4099 | $3.90 \times 10^{-1}$ | 12037 |
| 8 | 10 | $1.49 \times 10^{-2}$ | 1693 | 1.35 | 22 |
| | 15 | $3.42 \times 10^{-2}$ | 1630 | 1.35 | 21 |
| | 20 | $1.91 \times 10^{-2}$ | 1630 | 1.35 | 20 |
| | 25 | $1.57 \times 10^{-2}$ | 4914 | 1.35 | 22 |
| | 30 | $5.60 \times 10^{-3}$ | 4640 | 1.35 | 25 |
